# Cystathionine β-synthase is involved in cysteine biosynthesis and H_2_S generation in *Toxoplasma gondii*

**DOI:** 10.1038/s41598-020-71469-x

**Published:** 2020-09-04

**Authors:** Carolina Conter, Silvia Fruncillo, Carmen Fernández-Rodríguez, Luis Alfonso Martínez-Cruz, Paola Dominici, Alessandra Astegno

**Affiliations:** 1 Department of Biotechnology, University of Verona, Strada Le Grazie 15, 37134 Verona, Italy; 2 Center for Cooperative Research in Biosciences (CIC bioGUNE), Basque Research and Technology Alliance (BRTA), Bizkaia Technology Park, Building 801A, 48160 Derio, Spain; 3 Present Address: Manchester Institute of Biotechnology, University of Manchester, 131 Princess Street, Manchester, M1 7DN UK

**Keywords:** Enzyme mechanisms, Enzymes

## Abstract

Cystathionine β-synthase (CBS) catalyzes the condensation of serine and homocysteine to water and cystathionine, which is then hydrolyzed to cysteine, *α*-ketobutyrate and ammonia by cystathionine γ-lyase (CGL) in the reverse transsulfuration pathway. The protozoan parasite *Toxoplasma gondii,* the causative agent of toxoplasmosis, includes both CBS and CGL enzymes. We have recently reported that the putative *T. gondii* CGL gene encodes a functional enzyme. Herein, we cloned and biochemically characterized cDNA encoding CBS from *T. gondii* (TgCBS), which represents a first example of protozoan CBS that does not bind heme but possesses two C-terminal CBS domains. We demonstrated that TgCBS can use both serine and *O*-acetylserine to produce cystathionine, converting these substrates to an aminoacrylate intermediate as part of a PLP-catalyzed β-replacement reaction. Besides a role in cysteine biosynthesis, TgCBS can also efficiently produce hydrogen sulfide, preferentially via condensation of cysteine and homocysteine. Unlike the human counterpart and similar to CBS enzymes from lower organisms, the TgCBS activity is not stimulated by *S*-adenosylmethionine. This study establishes the presence of an intact functional reverse transsulfuration pathway in *T. gondii* and demonstrates the crucial role of TgCBS in biogenesis of H_2_S.

## Introduction

**Figure 1:**
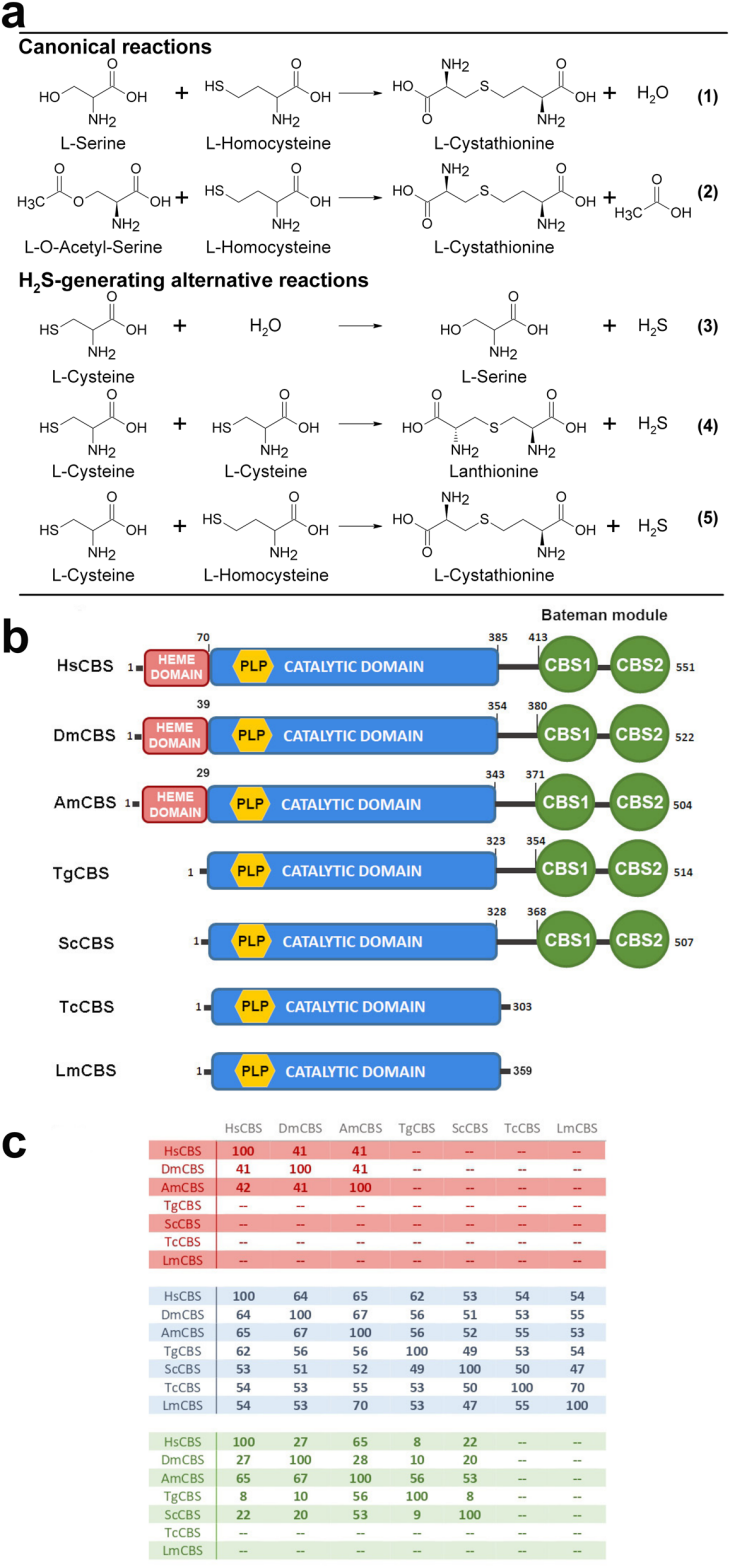
Reactions catalyzed by CBS and overall architecture and sequence similarity of CBSs from different organisms. (**a**) Reactions catalyzed by CBS characterized in this study. (**b**) Domain distribution in CBSs from *Homo sapiens* (HsCBS), *Drosophila melanogaster* (DmCBS), *Apis mellifera* (AmCBS), *Toxoplasma gondii* (TgCBS), *Saccharomyces cerevisiae* (ScCBS), *Trypanosoma cruzi* (TcCBS) and *Leishmania major* (LmCBS). (**c**) Sequence identity between the CBS enzymes shown in (**b**). The numbers indicate the amino acid sequence identity (expressed in percentage) between each CBS protein and its homologs in each domain (colored as in panel **b**).

The reverse transsulfuration pathway is well described in higher organisms^1–5^, where it is responsible for the generation of l-cysteine (l-Cys) from methionine via the intermediates homocysteine (l-Hcys) and cystathionine (l-Cth). The pathway includes two key pyridoxal 5′-phosphate (PLP)-dependent enzymes, namely cystathionine β-synthase (CBS, EC 4.2.1.22) and cystathionine γ-lyase (CGL, EC 4.4.1.1). CBS catalyzes the first step of the pathway where the –OH group of serine (l-Ser) is replaced by the thiol of l-Hcys in a β-replacement reaction to form l-Cth (reaction 1, Fig. 1a). l-Cth is then cleaved into l-Cys, ammonia, and *α*-ketobutyrate by CGL.

Besides playing crucial roles in metabolism of sulfur-containing amino acid, CBS is a prominent enzyme for the production of hydrogen sulfide (H_2_S) which is considered a critical signaling molecule involved in modulating physiological responses in various diseases and as a modulator of oxidative stress^6^. CBS can produce H_2_S via different routes such as β-elimination of l-Cys (reaction 3, Fig. 1a), β-replacement of two molecules of L-Cys (reaction 4, Fig. 1a) or via the condensation of L-Cys with L-Hcys (reactions 5, Fig. 1a), with reaction 5 considered the main mechanism by which the enzyme produces H_2_S^7,8^.

CBS is a PLP-requiring enzyme with complex domain organization and regulatory mechanisms that are not conserved across eukaryotes^9^ (Fig. 1b). The well-studied human CBS is a homotetrameric enzyme and each monomer consists of three distinct domains: the N-terminal domain which binds heme^10,11^, the highly conserved central fold type II PLP catalytic domain^12,13^, and the C-terminal domain which contains two CBS structural motifs (commonly referred to as the Bateman module) involved in the regulation of activity and oligomerization of various CBS enzymes^1,14–16^. CBS is the only known PLP-dependent protein that binds heme^17^. However, the role of heme in CBS is not fully understood, even if functions in redox sensing and/or enzyme stability and folding have been suggested^10,18–20^. Notably, the heme-domain has been found only in CBS from higher organisms^1–3,5^, while CBS enzymes from lower eukaryotic organisms (e.g., *S. cerevisiae* and *T. cruzi*^21^) lack this domain, thus implying that it is not necessary for enzymatic activity (Fig. 1b). Along with this, the deletion of this domain has no significant effect on enzyme activity^22^. The C-terminal domain is present in the majority of eukaryotic CBS enzymes; however, only mammalian CBS enzymes appear to be regulated by *S*-adenosylmethionine (SAM)^5,23–25^. Recently, the three-dimensional structure of full-length human CBS revealed the presence of two conformations, basal and activated, in the human enzyme^1, 2^. Without SAM, CBS is in a basal conformation with the CBS C-terminal domains occluding the entrance to the catalytic cavity. Binding of SAM induces rotation of the two CBS motifs, resulting in reduced interactions with the catalytic core and leading to an open, activated conformation. Strikingly, this conformation is structurally close to those found in the drosophila and honeybee enzymes ^3,5^, which are constitutively active, are not soluble upon removal of the C-terminal domain, and do not bind SAM. Yeast CBS, whose three-dimensional structural knowledge is limited to the catalytic core^26^, can bind SAM but its activity is not significantly regulated by the binding of the molecule^9^.

The protozoan parasite *T. gondii,* the etiological agent of toxoplasmosis, includes enzymes of the reverse transsulfuration pathway CBS and CGL. We have recently demonstrated that *T. gondii* CGL is functional, being able to break down l-Cth into l-Cys^27^. On the other hand, *T. gondii* CBS has not been functionally characterized. Interestingly, contrary to CBSs from other protozoans (e.g., *L. major* and *T. cruzi*) which lack the C-terminal Bateman module^21,28,29^, *T. gondii* CBS possesses a C-terminal region composed of two CBS domains, suggesting that, similarly to higher organisms, it might be allosterically regulated by adenosine derivatives. To this end, the biochemical and kinetic characterization of TgCBS could provide valuable information to obtain deeper insights on regulation of CBS enzymes in eukaryotes. Moreover, *T. gondii* CBS does not possess heme, making it a useful model to probe the catalytic mechanism via spectroscopic analysis (Fig. 1b).

Herein, we report a detailed steady-state characterization of the Toxoplasma CBS-catalyzed reactions along with spectroscopic characterization of enzyme-substrate intermediates. We found that the Toxoplasma enzyme possesses CBS activity not only with L-Ser, but also with activated serine (l-*O*-acetyl-serine, l-OAS) and produces l-Cth via aminoacrylate as an intermediate of the β-replacement reaction. TgCBS is also strongly implicated in the production of H_2_S, being highly efficient in catalyzing the β-replacement of l-Cys with l-Hcys. Importantly, the enzyme does not respond to SAM stimulation. These results strongly support previously published studies, indicating that the reverse transsulfuration pathway is fully functional in *T. gondii* and that CBS has a crucial role in the biogenesis of H_2_S.

## Results

### Properties of recombinant TgCBS

We identified the Toxoplasma gene likely to encode the CBS enzyme by searching the Toxoplasma genome database (https://toxodb.org/toxo/). The *T. gondii* (*strain ME49*) genome contains a single-copy gene for CBS (TGME49_259180) encoding a multimodular protein of 514 amino acids (55.8 kDa) that lacks the N-terminal heme-binding motif preceding the catalytic core domain observed in humans but possesses the Bateman module. Moreover, TgCBS also possesses the oxidoreductase (CysXXCys) motif present in the catalytic core of human CBS. Notably, CBSs from other protozoa, e.g. *T. cruzi* and *L. major*, lack the N-terminal heme domain, the C-terminal Bateman module and the oxidoreductase motif. The amino acid sequence similarity of TgCBS with its homologs varies significantly along the different domains of its polypeptide chain. For example, TgCBS maintains high identity with the catalytic core of eukaryotic CBSs (e.g. ~ 62%, 56%, 56%, 49%, 53% and 54% identity with respect to humans, fly, honeybee, yeast, Trypanosoma, and Leishmania, respectively), but shows a low similarity with the regulatory domain of these proteins [e.g. ~ 8%, 10%, and 8% with respect to humans, fly and yeast CBSs, respectively (Trypanosoma and Leishmania lack this domain)] (Fig. 1c). These distinct features determine the affinity for the different substrates and regulatory molecules (i.e. SAM in HsCBS), and thus the preferent catalytic activity and regulatory mechanisms across the organisms.

**Figure 2:**
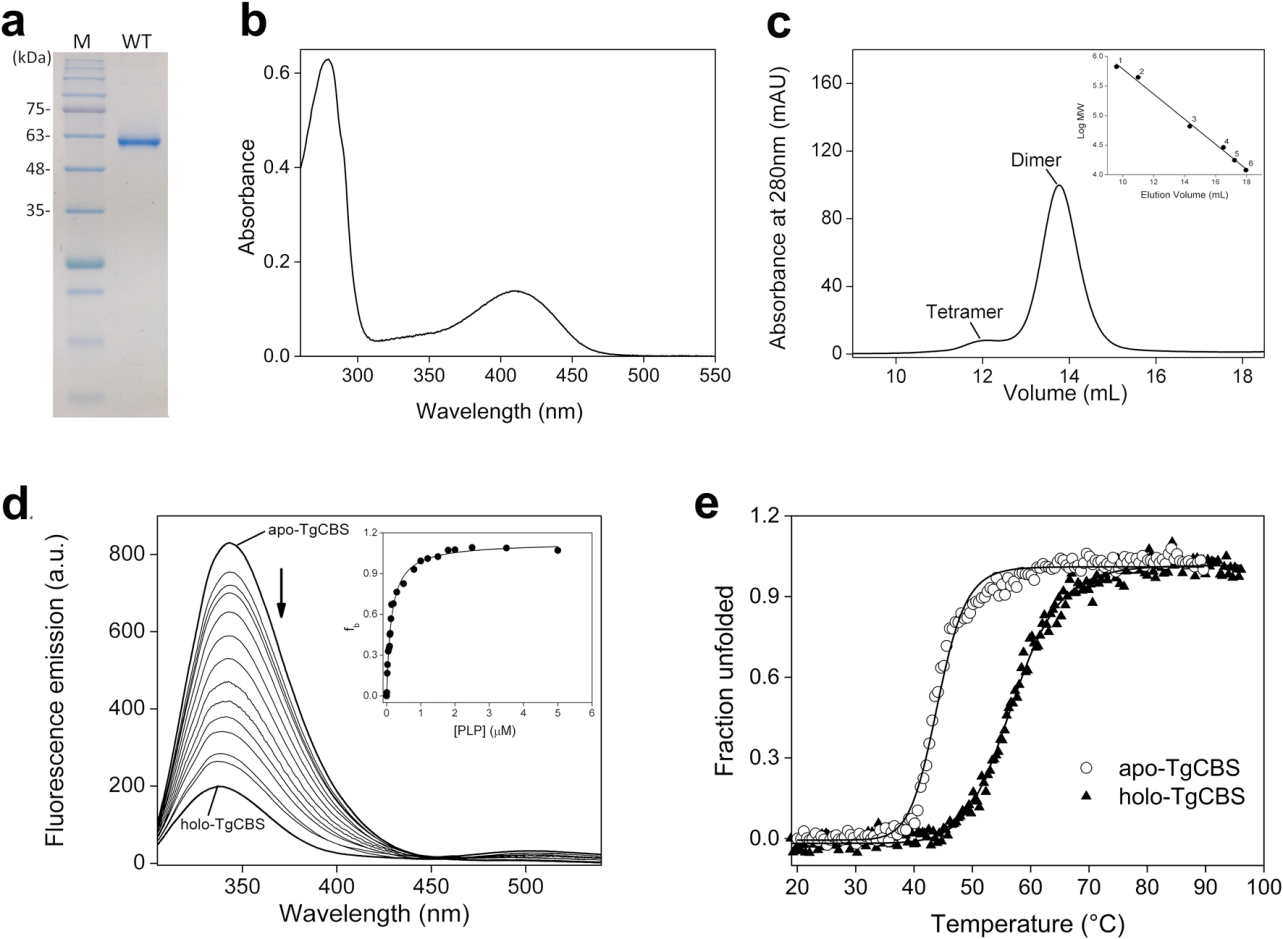
Properties of TgCBS. (**a**) 12% SDS-PAGE analysis of purified recombinant TgCBS. Lane M, protein marker. (**b**) UV-visible absorption spectrum of 15 µM purified TgCBS recorded in 20 mM sodium phosphate buffer pH 8.5. (**c**) Gel filtration chromatography of TgCBS at 1 mg/mL using a Superdex 200 10/30 GL column in 20 mM sodium phosphate, 150 mM NaCl buffer pH 8.5. *Inset,* calibration curve of logarithm of the molecular weight versus elution volumes (Ve). The standard proteins used were: (1) thyroglobulin; (2) apoferritin; (3) albumin bovine serum; (4) carbonic anhydrase; (5) myoglobin; (6) cytochrome c. (**d**) Representative fluorescence titration of apo-TgCBS (1 μM) with PLP (0.01–5 μΜ). The fluorescence emission upon excitation at 295 nm was determined 5 min after each addition of PLP in 20 mM sodium phosphate buffer pH 8.5. The *K*_d_ value is determined by fitting the fraction of bound PLP (f_b_) to a hyperbolic equation (inset) and represents a mean value ±  s.e.m. of three independent measurements. F_b_ is calculated as follows: f_b_ = (F − F_0_)/(F_max_ − F_0_), where F_0_ is the emission fluorescence of the protein at zero PLP concentration, F_max_ is the value at saturating PLP concentration and F is the value as a function of PLP (x-axis) concentration. (**e**) Thermal denaturation of 0.2 mg/mL apo- (open circles) and holo-TgCBS (solid triangles) recorded following ellipticity signal at 222 nm in 20 mM sodium phosphate buffer pH 8.5.

TgCBS was overexpressed in *E. coli* and purified as His-tagged protein with purity higher than 95% as judged by SDS-PAGE (Fig. 2a). The recombinant protein was yellow and exhibited a UV-visible absorption spectrum with a major peak at 410 nm characteristic of the ketoenamine tautomer of the internal aldimine (PLP bound to active site Lys56) (Fig. 2b)^30^. No evidence of a heme group was found as illustrated by the absence of Soret band at 430 nm in the absorption profile. In solution, the protein was predominantly present as a dimer (~ 97 kDa) with some high order oligomers [e.g., tetramer (~ 234 kDa), Fig. 2c], in accordance with a monomer molecular mass of ~ 56 kDa. TgCBS binds ~ 1 mol of PLP/mol of monomer with a *K*_d_ value for PLP of 0.13 ± 0.01 µM, as deduced by fluorescence titrations of apo-TgCBS with PLP (Fig. 2d). Moreover, PLP affects the thermal stability of the enzyme. Compared to the sample containing apo-protein, the melting temperature (*T*_*m*_) of TgCBS with PLP increased from 44 ± 1 °C to 56 ± 1 °C. (Fig. 2e).

### Steady-state characterization of TgCBS

We determined the steady-state kinetic parameters for TgCBS in the canonical and H_2_S-generating alternative reactions described in Fig. 1a.

#### CBS canonical reactions

The steady-state kinetic parameters of TgCBS in the canonical reactions were determined by applying a highly sensitive continuous assay based on recombinant cystathionine beta-lyase (CBL) from *Corynebacterium diphtheriae* produced in our laboratory^31,32^ and on commercial lactate dehydrogenase (LDH) as coupling enzymes, following a method described in Ref.^33^ (see “Materials and methods”). Nascent l-Cth is captured by CBL and converted to l-Hcys, NH_3_, and pyruvate, which is then detected by LDH assay (decrease in absorbance at 340 nm, reflecting the oxidation of NADH by LDH) (Supplementary Fig. S1a). The continuous nature of the assay avoids accumulation of the l-Cth product, which can compete with l-Ser for free enzyme, therefore preventing the phenomenon of product inhibition.

To test the usefulness of the coupled-coupled enzyme assay to detect CBS activity, we first investigated the kinetic parameters of our recombinant CBL in catalyzing the β-elimination of l-Cth to pyruvate, l-Hcys and NH_3_ (*k*_cat_ = 93 ± 2 s^−1^, K_m_ = 0.8 ± 0.1 mM, *k*_cat_/K_m_ = 116 mM^−1^ s^−1^) and of l-Ser to pyruvate and NH_3_ (*k*_cat_ = 1.2 ± 0.2 s^−1^, K_m_ = 7.5 ± 1.4 mM, *k*_cat_/K_m_ = 0.16 mM^−1^ s^−1^) under the conditions used for the CBS coupled-coupled assay. The obtained values agree with those previously published by our laboratory^31, 32^. Importantly, the catalytic efficiency of CBL toward l-Ser was < 0.2% compared to l-Cth, and thus this low activity does not interfere with the accuracy of the assay. Next, the assay was optimized for the amount of auxiliary enzymes by measuring the NADH oxidation rate in standard assay mixtures containing different concentrations of CBL or LDH. It was necessary to use 1.5 µM CBL and 2 µM LDH in the coupled-coupled assay because these concentrations are each well into the plateau region of coupling enzymes for the range of TgCBS concentrations assayed (dependence of NADH oxidation rate in the coupled-coupled assay was found to be linear in the 0.2–2 μM TgCBS concentration range) (Supplementary Fig. S1b).

Initially, the CBS assay was performed with constant substrate concentrations (10 mM l-Ser and 0.8 mM l-Hcys) from pH 5.5 to 9.5 and the optimum activity was observed around pH 9 (Supplementary Fig. S2a). Thus, pH 9 was used for further CBS enzymatic characterization. Moreover, we evaluated residual activity of purified TgCBS after thermal stresses of 10 min at temperatures ranging from 30 to 70 °C. We found a *T*_50_ (half-inactivation temperature) of 44.6 ± 0.3 (Supplementary Fig. S2b).

**Figure 3:**
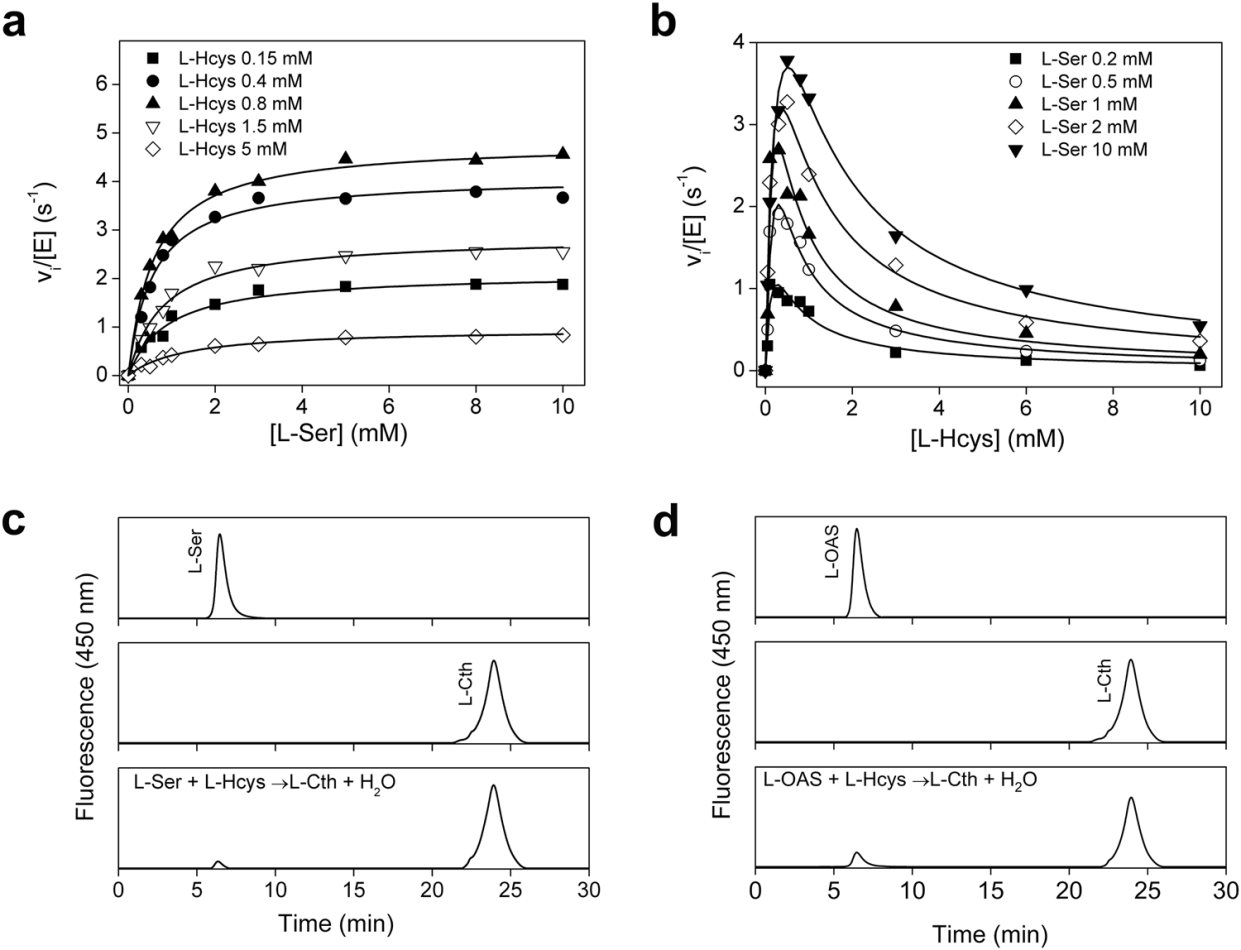
Canonical reactions. (**a**,**b**) Representative steady-state initial velocity kinetics for TgCBS showing the dependence of the reaction on l-Ser and l-Hcys concentrations. (**a**) The concentration of l-Ser was varied at fixed concentration of l-Hcys. (**b**) The concentration of l-Hcys was varied at fixed concentration of l-Ser. Data fit were performed according to Eq. (1). (**c**,**d**) Analysis of reaction products using reverse phase HPLC. (**c**) Pure standard l-Ser (top), pure standard l-Cth (middle), and product obtained following a 2 h incubation of TgCBS (1.5 µM) with 1 mM l-Ser and 0.8 mM l-Hcys (bottom). (**d**) Pure standard l-OAS (top), pure standard l-Cth (middle) and product obtained following a 2 h incubation of TgCBS (1.5 µM) with 1 mM l-OAS and 0.8 mM l-Hcys (bottom).

Experimental data for the condensation of l-Ser and l-Hcys to l-Cth catalyzed by TgCBS are shown in Fig. 3a,b. The phenomenon of substrate inhibition was negligible for L-Ser and evident for l-Hcys as pointed out by the decrease in initial velocity at high substrate concentrations. Both human CBS^34^ and yeast CBS^4,33^ are characterized by substrate inhibition by l-Hcys. We collected a large data set as necessary for a bi-substrate system and the data were fit according to Eq. (1), which includes a K_i_ value representing the inhibition constant for substrate inhibition by l-Hcys^4,33^. The kinetic parameters are summarized in Table 1. TgCBS showed a *k*_cat_ for condensation of l-Ser and l-Hcys of 6.3 ± 0.4 s^−1^ and K_m_ values for l-Ser and l-Hcys of 0.42 ± 0.04 mM and 0.23 ± 0.03 mM*,* respectively (Table 1). At high concentration of L-Hcys, the enzyme displayed substrate inhibition with a K_i_ value of 1.0 ± 0.1 mM (Table 1). Thus, the parasitic enzyme is significantly inhibited by l-Hcys which can either compete directly with the l-Ser substrate for the binding site on the free enzyme (E) or bind to the enzyme-substrate complex (E-l-Ser) before releasing water.

**Table 1:** Steady-state kinetic parameters of TgCBS for canonical reactions.

Kinetic parameter	TgCBS
**l-Ser + l-Hcys → l-Cth + H**_**2**_**O**	
*k*_cat_ (s ^ −1^)	6.3 ± 0.4
K_m_^L-Ser^ (mM)	0.42 ± 0.04
K_m_^L-Hcys^ (mM)	0.23 ± 0.03
*k*_cat_/K_m_^L-Ser^ (mM^−1^ s^−1^)	15 ± 2
*k*_cat_/K_m_^L-Hcys^ (mM^−1^ s^−1^)	27 ± 5
K_i_^L-Hcys^ (mM)	1.0 ± 0.1
**l-OAS + l-Hcys → l-Cth + H**_**2**_**O**	
*k*_cat_ (s^−1^)	5.5 ± 0.1
K_m_^L-OAS^ (mM)	1.3 ± 0.2
K_m_^L-Hcys^ (mM)	0.20 ± 0.05
*k*_cat_/K_m_^L-OAS^ (mM^−1^ s^−1^)	4.2 ± 0.7
*k*_cat_/K_m_^L-Hcys^ (mM^−1^ s^−1^)	28 ± 7
K_i_^L-Hcys^ (mM)	1.4 ± 0.2

Reactions were carried out in MBP (see “Materials and methods”) buffer pH 9 containing 0.2 mM NADH, 2 μM LDH, 1.5 μM CBL, and 0.1–10 mM l-Ser (or 1–100 mM l-OAS), 0.1–10 mM l-Hcys and 0.2–2 μM TgCBS at 37 °C. Data were fit to Eq. (1). Data are mean ± s.e.m.

Further analysis of the activity of CBS was performed to evaluate if TgCBS also synthetizes l-Cth via the β-replacement reaction of l-OAS and l-Hcys (reaction 2 in Fig. 1a). Interestingly, TgCBS can also act on l-OAS, even if the catalytic efficiency was ~ threefold lower compared to l-Ser, as it is affected by higher K_m_ values. Substrate inhibition was also observed for l-OAS-dependent CBS activity at high concentrations of l-Hcys (K_i_ = 1.4 ± 0.2 mM) (Table 1).

The ability of TgCBS to use both l-Ser and l-OAS to produce l-Cth was further supported by analysis of reaction products using reverse phase HPLC in combination with ortho-phthaldialdehyde (OPA) derivatization (Fig. 3c,d). The retention times of l-Ser, l-OAS, and l-Cth commercial standards were 6.4 min, 6.5 min and 23.9 min, respectively (Fig. 3c,d). Importantly, HPLC detected only l-Cth upon incubation of TgCBS with l-Hcys and either l-Ser (Fig. 3c) or l-OAS (Fig. 3d).

Since human CBS is allosterically activated by SAM, we also investigated the enzymatic activity of TgCBS in the presence of SAM. However, no significant response to SAM was observed in the 0–0.5 mM SAM concentration range (Supplementary Table S1).

#### *Alternative CBS ability to generate H*_*2*_*S*

The enzymatic ability of TgCBS to produce H_2_S in the presence of l-Cys alone or with l-Hcys was also analyzed. The generation of H_2_S catalyzed by TgCBS was monitored by the lead acetate method^7^.

**Figure 4:**
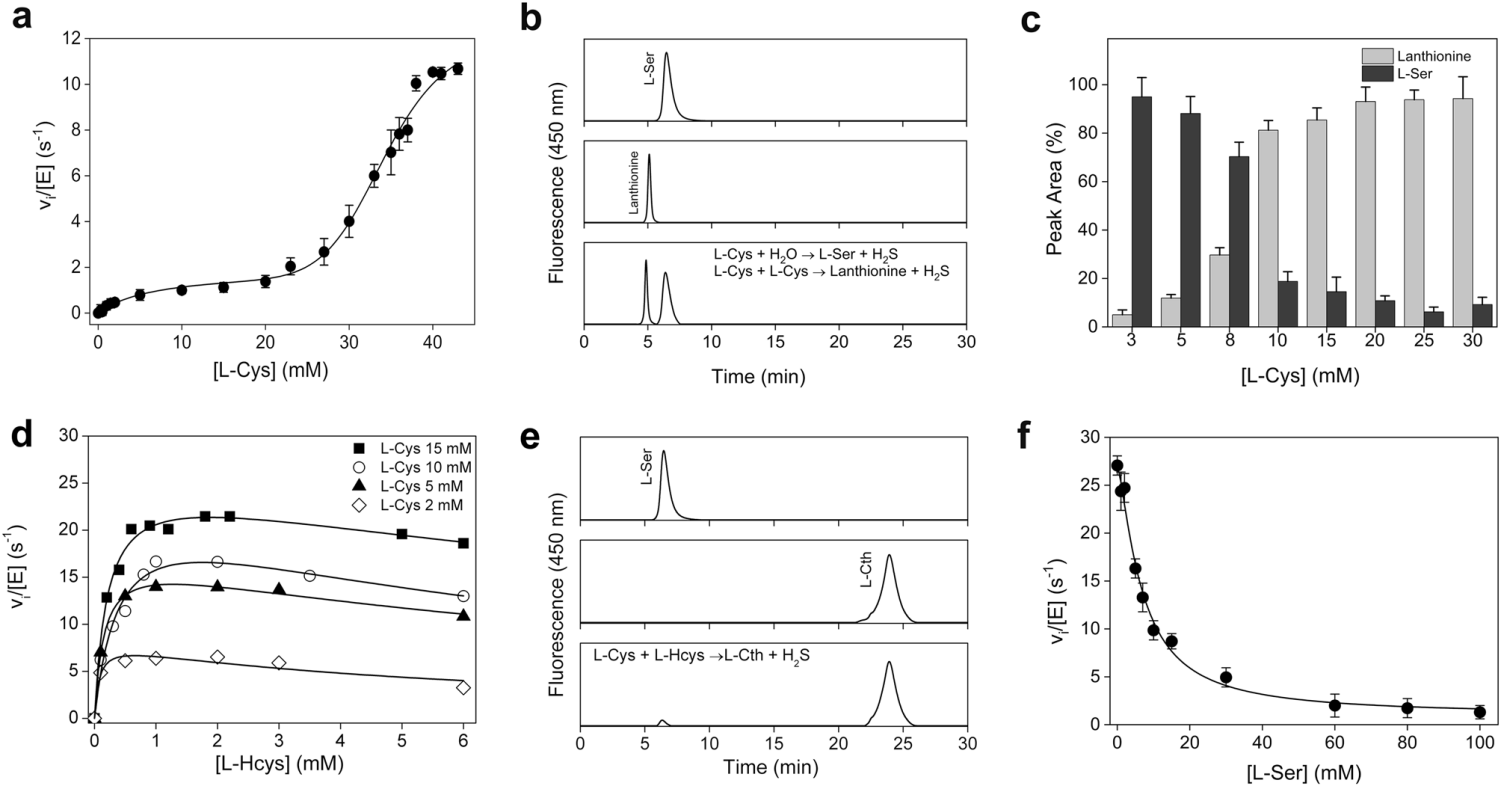
H_2_S alternative reactions. (**a**–**c**) Ability of TgCBS to use l-Cys. (**a**) Steady-state initial velocity kinetic for TgCBS showing the dependence of the reaction on l-Cys concentrations. Data fit was performed according to Eq. (2) and the kinetic parameters obtained from the plot are shown in Table 2. Each data point represents the mean ± s.e.m. of at least three independent experiments. (**b**) Representative analysis of reaction products using reverse phase HPLC. Pure standard l-Ser (top), pure standard lanthionine (middle) and product obtained following a 2 h incubation of TgCBS (1.5 µM) with 8 mM l-Cys (bottom). (**c)** l-Ser and lanthionine production upon incubation of TgCBS (1.5 µM) with increasing concentrations of l-Cys in 50 mM Hepes pH 7.4. Each bar represents the mean ± s.e.m. of four independent experiments. (**d**–**f**) Condensation of l-Cys and l-Hcys. (**d**) Representative steady-state initial velocities for TgCBS at various concentrations of l-Hcys, while keeping l-Cys at different fixed concentrations. Data analysis and fitting were performed using Eq. (1). (**e**) Representative analysis of reaction products using reverse phase HPLC. Pure standard l-Ser (top), pure standard l-Cth (middle) and product obtained following a 2 h incubation of TgCBS (1.5 µM) with 1 mM l-Cys and 0.8 mM l-Hcys (bottom). (**f**) Substrate competition assay in H_2_S-forming TgCBS condensation of l-Cys and l-Hcys in the presence of increasing l-Ser concentration (0–100 mM) and fixed l-Cys (20 mM) and l-Hcys (0.8 mM) concentrations. Each data point represents the mean ± s.e.m. of three independent experiments.

Experimental data for H_2_S production from l-Cys followed a markedly biphasic profile (Fig. 4a) which is consistent with the fact that the production of H_2_S from l-Cys arises from both unimolecular (β-elimination of l-Cys to generate l-Ser, reaction 3, Fig. 1a) and bimolecular (β-replacement of 2 mol of l-Cys to generate lanthionine, reaction 4 Fig. 1a) reactions. This allowed us to deconvolute the kinetic parameters for reactions 3 and 4, respectively (Table 2) by using Eq. (2) with *v*_L-ser_ defined in Eq. (3) and *v*_lanth_ defined in Eq. (4), following the procedure described by Singh et al.^8^. The active-site of CBS can accommodate two substrates, i.e. l-Ser and l-Hcys in the canonical reaction, in the so-called site-1, where the external aldimine with l-Ser is formed, and site-2, where l-Hcys is docked for reaction with α-aminoacrylate to generate l-Cth. Thus, L-Cys can bind to both sites. TgCBS showed a ~ sevenfold higher K_m_ for binding of the second mol of l-Cys (34 ± 3 mM) with respect for binding of l-Cys to site 1 (4.7 ± 0.9 mM). The *k*_cat_ for condensation of two molecules of l-Cys (reaction 4, Fig. 1a) is ~ fivefold higher than for the β-elimination of l-Cys (reaction 3, Fig. 1a) (Table 2).

**Table 2:** Steady-state kinetic parameters of CBS for H_2_S-generating reactions.

Kinetic parameter	TgCBS
**l-Cys + H**_**2**_**O → l-Ser + H**_**2**_**S**	
*k*_cat_ (s^−1^)	2.6 ± 0.3
K_m_ (mM)	4.7 ± 0.9
*k*_cat_/K_m_ (mM^−1^ s^−1^)	0.6 ± 0.2
K_i_ (mM)	36 ± 4
**l-Cys + l-Cys → Lanthionine + H**_**2**_**S**	
*k*_cat_ (s^−1^)	12 ± 2
K_m_ (mM)	34 ± 3
*k*_cat_/K_m_ (mM^−1^ s^−1^)	0.4 ± 0.1
n	3.0 ± 0.5
**l-Cys + l-Hcys → l-Cth + H**_**2**_**S**	
*k*_cat_ (s^−1^)	29 ± 1
K_m_^L-Cys^ (mM)	5.6 ± 0.8
K_m_^L-Hcys ^(mM)	0.46 ± 0.09
*k*_cat_/K_m_^L-Cys^ (mM^−1^ s^−1^)	5.2 ± 0.9
*k*_cat_/K_m_^L-Hcys^ (mM^−1^ s^−1^)	63 ± 14
K_i_^L-Hcys^ (mM)	7.1 ± 0.4

H_2_S-production activity experiments were carried out in 50 mM Hepes pH 7.4 at 37 °C. Data are mean ± s.e.m.

The ability of TgCBS to catalyze both the β-elimination and the condensation reactions starting from l-Cys was further confirmed via reverse phase HPLC (Fig. 4b). The comparison of the HPLC profiles obtained following incubation of TgCBS in the presence of l-Cys with those of l-Ser and lanthionine commercial standards allowed the identification of both reaction products l-Ser and lanthionine. Interestingly, quantitative analysis of l-Ser and lanthionine production in the presence of increasing concentration of l-Cys confirmed that the production of l-Ser prevails at low concentrations of l-Cys while at higher concentrations of l-Cys the predominant product is lanthionine (Fig. 4c).

The formation of H_2_S significantly increased when TgCBS catalyzed the condensation reaction of l-Cys and l-Hcys (reaction 5, Fig. 1a) and the *k*_cat_ value for the reaction was ~ 11-fold higher than that for l-Cys alone (Fig. 4d, Table 2). Importantly, no H_2_S formation from l-Hcys alone was detected by the lead acetate assay. In accordance with kinetic data, HPLC analysis of reaction products obtained upon incubation of TgCBS with l-Hcys and l-Cys resulted in a main fluorescence peak corresponding to l-Cth and one minor peak ascribable to l-Ser (Fig. 4e). Thus, TgCBS produces H_2_S preferentially via β-replacement of l-Cys with l-Hcys than β-elimination of l-Cys or β-replacement of two molecules of l-Cys.

Of note, the *k*_cat_ value obtained for the β-replacement of l-Ser and l-Hcys (6.3 ± 0.4 s^−1^, reaction 1, Fig. 1a)  is ~ fivefold lower than that for the condensation of l-Cys and l-Hcys (29 ± 1 s^−1^, reaction 5, Fig. 1a). Since l-Ser and l-Cys likely coexist in the cell at physiological conditions, we investigated the β-replacement reaction of l-Cys with l-Hcys in the presence of l-Ser as a competing substrate. Increasing concentrations of l-Ser resulted in a decrease in H_2_S production, indicating that l-Ser inhibits the condensation of l-Cys with l-Hcys (Fig. 4f). The IC_50_ value, i.e. the concentration of l-Ser at which the H_2_S production and therefore the activity of TgCBS was half-maximal, was 6.7 ± 1.3 mM.

### Spectroscopic analysis in the presence of substrates, products and analogs

**Figure 5:**
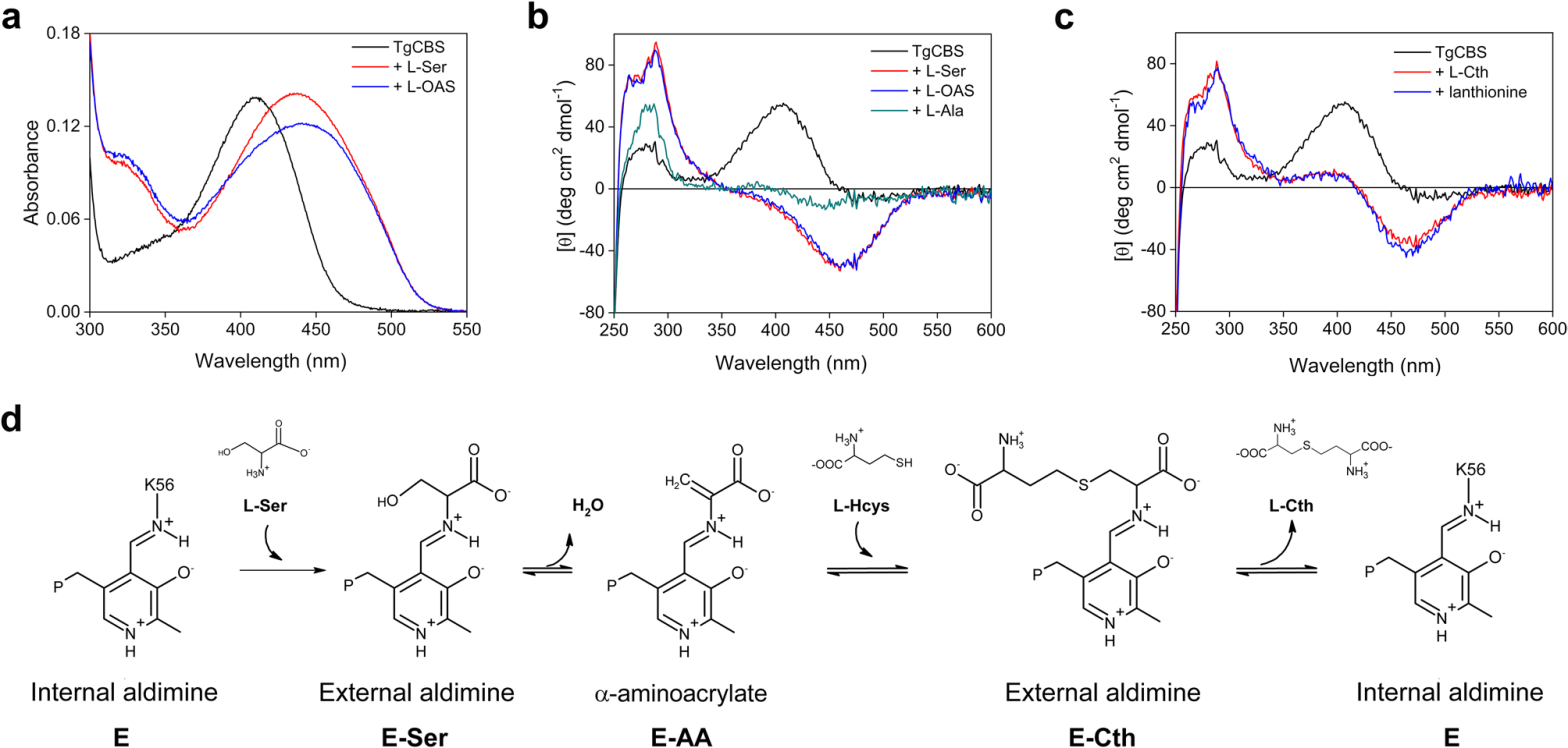
Spectra of TgCBS in the presence of substrates, analogs, and products. (**a**) Absorbance spectra of 15 µM TgCBS in the presence of the substrates 10 mM l-Ser or 10 mM l-OAS. (**b**) CD spectra of 1 mg/mL TgCBS alone and in the presence of 10 mM l-Ser, or 10 mM l-OAS, or 25 mM l-Ala. (**c**) CD spectra of 1 mg/mL TgCBS alone and in the presence of the products 4 mM l-Cth or 3 mM lanthionine. (**d**) Reaction intermediates formed in the active-site of TgCBS upon reaction with the substrates l-Ser and l-Hcys.

The absence of heme in TgCBS (Fig. 1b) offered the opportunity to spectroscopically investigate the intermediates in reactions catalyzed by TgCBS. Addition of l-Ser or l-OAS to the TgCBS solution resulted in the disappearance of the 410 nm-peak and the appearance of a major band centered at 440 nm together with an increase at 330 nm (Fig. 5a). The 440–460 nm band is usually ascribed to the aminoacrylate species^30^. However, since the attribution of the band at 440 nm to the aminoacrylate species in the UV-visible spectra of TgCBS may not be straightforward, we analyzed the PLP-dependent changes elicited by l-Ser and l-OAS by CD spectroscopy. TgCBS alone exhibited a pronounced positive CD peak at 410 nm and a modest band at 280 nm. The addition of l-Ser or l-OAS resulted in a negative band at 460 nm and in an increased signal of the positive band at 280 nm (Fig. 5b). These spectra allowed us to assign the peak at 440–460 nm to the aminoacrylate intermediate. Based on these data, the second absorption peak at 320–330 nm can be assigned to a different tautomer, i.e., the enolimine tautomer of the aminoacrylate species.

Addition of the analog l-alanine (l-Ala) to TgCBS resulted in a shift from a positive 410 nm CD band to a modest negative band centered at 430 nm, in accordance with the conversion of internal to external aldimine (Fig. 5b). No changes in both the absorption and CD spectra were observed following addition of l-Hcys to the enzyme. Thus, l-Hcys cannot form an external aldimine with TgCBS (data not shown).

**Figure 6:**
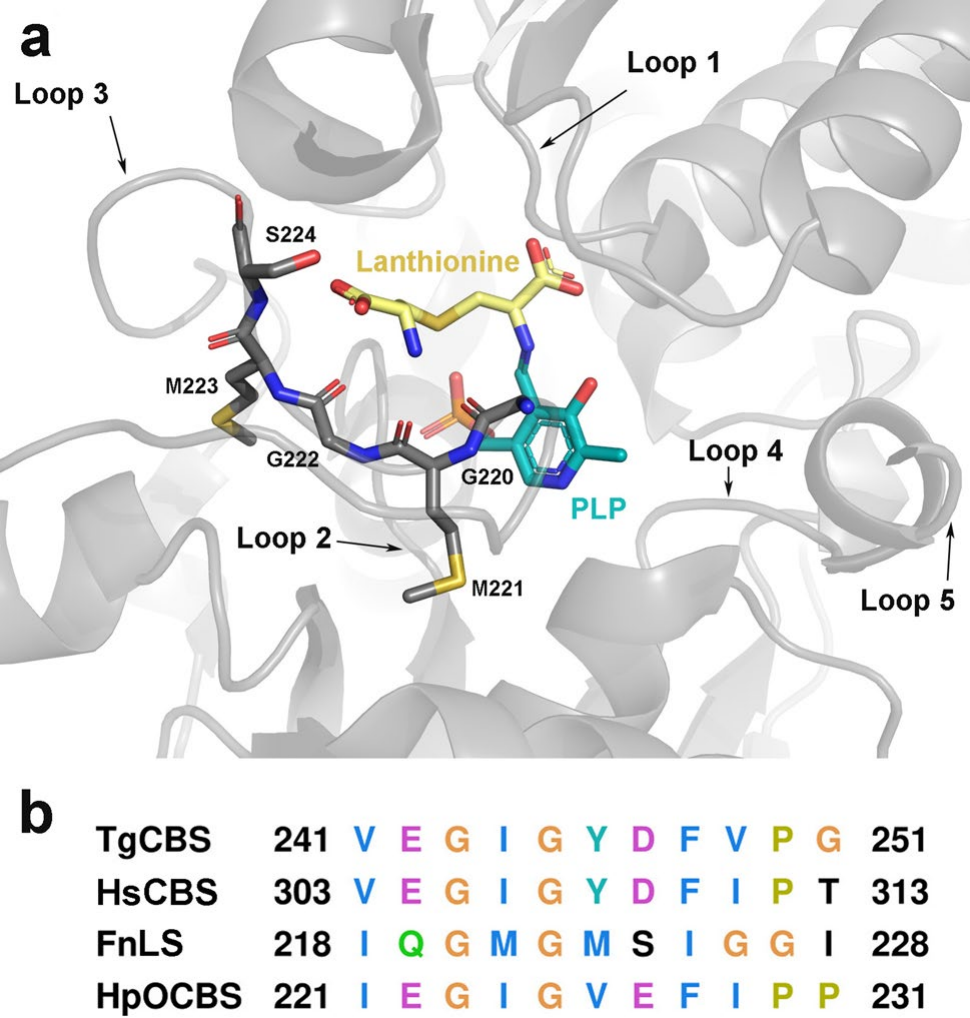
Lanthionine site in PLP-dependent related enzymes. (**a**) Ribbons and sticks representation of the lanthionine-PLP location at the catalytic site of lanthionine synthase from *Fusobacterium nucleatum*, whose structure represents one of the few examples with bound lanthionine (the coordinates are extracted from PDB ID entry 5XEM^35^). Similar to CBSs, the catalytic site of lanthionine synthase includes five conserved loops/blocks configuring the catalytic site. Loop 1, also known as the “asparagine loop”^36^, is known to interact with the first substrate involved in the β-replacement reaction, e.g. l-OAS^37^ or l-Ser^5^, and also helps stabilize the aminoacrylate intermediate; Loop-1 contains a conserved serine (S82 in yeast CBS^26^, S84 in TgCBS) that has been postulated to interact with the second substrate, l-Hcys during the canonical β-replacement^26,36^. Loop 2 anchors the phosphate moiety of PLP. Conserved Loop 3 (comprising residues 241VEGIGYD247 in TgCBS, highlighted in sticks in panel a) is postulated to interact with the second substrate, e.g. L-Hcys, in HpOCBS (residues 221IEGIGVE224) through two key residues, G245 and Y246^26^. Loop 4 and Loop 5 contribute to stabilize the orientation of PLP through interactions with the pyridine ring. (**b**) Partial sequence alignment of loop-3 in *Toxoplasma gondii* cystathionine β-synthase (TgCBS); human cystathionine β-synthase (HsCBS); *Fusobacterium nucleatum* lanthionine synthase (FnLS)^35^; *Helicobacter pylori* *O*-acetylserine-dependent CBS (HpOCBS)^36^.

We also measured the CD spectra of TgCBS in the presence of the product l-Cth to evaluate the reversibility of the CBS canonical reaction. The reaction with l-Cth caused the appearance of a pronounced negative peak at 460 nm and a broader positive peak at 400 nm. Moreover, an increase in the 280 nm band was observed (Fig. 5c). These changes are ascribable to the formation of an aminoacrylate intermediate (Fig. 5d), thus indicating partial reversibility of the reaction. This partial reversibility was also evident for the β-replacement reaction of two molecules of l-Cys (Fig. 5c). Binding of lanthionine to the enzyme (at the putative site described in Fig. 6) resulted in CD spectra that were comparable to those seen in the presence of l-Cth and led to the detectable accumulation of aminoacrylate intermediate at 460 nm.

## Discussion

Despite the major importance of redox balance in survival of parasites and the role that cysteine production has in redox homeostasis, to date knowledge about the cysteine biosynthetic pathway in *T. gondii* is still very limited^27^. The protozoan parasite includes both the CBS and CGL enzymes of the reverse transsulfuration pathway. We have shown previously that the putative CGL gene encodes a functional enzyme in *T. gondii*^27^. Herein, we establish that the parasite possesses an intact reverse transsulfuration pathway by cloning and characterization of the gene encoding putative CBS.

CBS from *T. gondii* is the first protozoan CBS enzyme, in the literature, which has been shown to possess a CBS-pair in the C-terminal region. Moreover, as evident from this work and in line with amino acid sequence analysis, the purified enzyme has no heme group. The observed absence of heme in the fully active recombinant TgCBS excludes a role of a heme domain in catalytic activity of the enzyme.

Like other CBS studied, TgCBS has a two-site ping-pong mechanism. The hydroxyl group of the first substrate l-Ser is replaced by the thiol group of l-Hcys. Herein, to study the canonical activity of CBS we employed a CBL/LDH coupled-coupled continuous assay that was first used by Aitken and Kirsch^33^ to overcome some major limitations of the ^14^C-l-Ser endpoint assay predominantly used in the CBS literature^38^. Indeed, even if the radioactive assay is sensitive, the authors pointed out that it is affected by product inhibition due to the accumulation of l-Cth which can compete with l-Ser for free enzyme. Therefore, the kinetic parameters might be overestimated because of the binding of l-Cth to the l-Ser binding site.

The coupled-coupled enzyme assay was very sensitive, permitting reliable detection of CBS activity. TgCBS, unsurprisingly, possesses CBS activity to produce l-Cth via condensation of l-Ser with l-Hcys, but can also form l-Cth from l-OAS. The ability of TgCBS to use both l-OAS and l-Ser as substrates might suggest that TgCBS is well suited for a variety of physiological conditions for which the comparative levels of the two substrates are altered. However, at present, there is a definite lack of information regarding the concentration of the two substrates during the diverse developmental stages of the parasite. Notably, CBSs from other protozoa, e.g., *L. maj*or and *T. cruzi*, can also efficiently utilize both l-Ser and l-OAS^28,29^, while prokaryotic CBSs, e.g., *L. plantarum* and *B. subtilis*, possess specific l-OAS dependent CBS activity showing CBS activity only with l-OAS and not with l-Ser^39,40^. On the other hand, CBS activity with L-OAS has not been reported for human CBS. Of note, CBS enzymes from the above-cited protozoa and prokaryotes lack both heme and the C-terminal regulatory domain (Fig. 1b,c). Thus, the difference in substrate preferences could be related to the active-site pocket of CBSs since the accommodation of the acetyl group of l-OAS at the l-Ser-binding site can be hampered by the heme and/or C-terminal CBS-pair. However, this should not take place in CBSs lacking one or other of the domains.

As expected for a PLP-catalyzed β-replacement reaction, TgCBS converts both l-Ser and l-OAS to a stable aminoacrylate intermediate. The aminoacrylate intermediate is also formed from the product, l-Cth, demonstrating the partial reversibility of the reaction. Our spectroscopic data support the notion that canonical β-replacement reaction in TgCBS proceeds via the intermediates showed in Fig. 5d, in which deprotonation of the Cα proton of l-Ser (bound at site-1) is followed by β-elimination of water from the external aldimine forming the aminoacrylate, whose reaction with l-Hcys (bound at site-2) resulted in the l-Cth product and regeneration of the free enzyme in its internal aldimine form. Importantly, no change in the absorption or CD spectra were visible upon addition of l-Hcys to TgCBS, indicating that l-Hcys does not form an external aldimine with TgCBS. Thus, only aminoacrylate was detected both in the forward and reverse direction. The lack of spectroscopically detectable accumulation of external aldimine could imply that the α-proton abstraction is not rate-limiting. Rapid scanning stopped-flow measurements will be needed for a better description and identification of intermediates in the reaction of TgCBS with l-Ser and l-Hcys.

Contrary to the human enzyme, but similar to the yeast CBS, TgCBS does not require SAM for its activation, thus supporting the notion that the presence of a heme moiety and allosteric regulation by SAM may differentiate CBS enzymes from higher versus lower eukaryotes. The absence of SAM stimulation may be due to several reasons: for example, TgCBS may not possess a binding site for SAM (as suggested by the poor sequence identity (8%) between human CBS and TgCBS in the Bateman module, Fig. 1b,c), therefore being unable to bind the molecule, or it may bind SAM but exist in a “locked” conformation such that the binding of SAM cannot activate it. Interestingly, even if yeast CBS cannot be significantly activated by SAM, it has been reported to be able to bind the molecule^9^. We must await in progress data on the three-dimensional structure of TgCBS for a thorough interpretation of the active site differences between the parasitic, yeast and human enzymes.

In humans, CBS is known to play a major role in generating endogenous H_2_S. Like the mammalian homologue, the *T. gondii* CBS can decompose l-Cys as well as catalyze the condensation of l-Cys with l-Hcys to generate H_2_S. Remarkably, TgCBS catalyzes more efficiently the β-replacement reaction of l-Cys and l-Hcys than the β-elimination of l-Cys. This finding is not surprising since the primary reaction performed by CBS is the condensation of l-Ser with l-Hcys and supports the notion that the CBS active-site is adapted to accommodate l-Hcys which adds to the aminoacrylate intermediate to yield the product l-Cth. Moreover, comparison of the kinetic parameters of TgCBS with those of human CBS reveals that the *T. gondii* enzyme is more active than the human enzyme in catalyzing the condensation of l-Cys with l-Hcys leading to production of l-Cth and H_2_S (steady-state kinetic parameters for the condensation of l-Cys with l-Hcys for human CBS: *k*_cat_ 8.17 ± 0.22 s^−1^, K_m_ 4.31 ± 0.05 mM^41^). The catalytic mechanism of the l-Cys and l-Hcys β-replacement reaction is ping-pong corresponding to the canonical condensation of l-Ser and l-Hcys which produces l-Cth, except that in the alternative reaction H_2_S is produced instead of H_2_O. Notably, under substrate saturating conditions, the turnover number of this H_2_S-generating reaction (29 ± 1 s^−1^) is fivefold higher than that of the l-Cth-generating canonical reaction (6.3 ± 0.4 s^−1^),which could in part be due to the better leaving group of H_2_S compared to H_2_O. However, it is not known when the alternative reactions occur and how they are regulated in the cell. The intracellular levels of l-Cys and l-Hcys need to be tightly regulated. At intracellular concentrations of l-Cys and l-Ser, l-Ser is likely more abundant, and thus it is expected that the β-replacement of l-Ser with l-Hcys is preferred over condensation of l-Cys and l-Hcys. Along with this, l-Ser can inhibit the H_2_S-generating reaction as suggested by our in vitro competition assays. Thus, the ratio between l-Ser and l-Cys concentration could be a significant factor affecting the generation of H_2_S via TgCBS, as already suggested for human CBS^41^. However, since no information about the concentration of the l-Ser and l-Hcys in the pathogen is known, these suggestions deserve further investigations.

The above data strongly indicate that TgCBS is an H_2_S-generating enzyme. Interestingly, while in human also CGL is primarily responsible for biogenesis of H_2_S via the hydrolysis of l-Cys, CGL in Toxoplasma has small reactivity toward l-Cys ^27^. Thus, in the parasite, CGL is not likely a major source to generate H_2_S, while TgCBS is expected to be mainly involved in production of the gas. These differences in H_2_S biosynthesis via transsulfuration between the human and parasite enzymes could have far-reaching consequences for the design of potential enzyme parasite-specific inhibitors.

Analysis of the H_2_S-alternative reactions also revealed that, by decomposing l-Cys, TgCBS can generate the non-proteinogenic amino acid lanthionine, connecting this metabolite to sulfur metabolism and TgCBS. While in humans lanthionine has been proposed as a reliable and stable marker of H_2_S synthesis, the presence and fate of lanthionine in Toxoplasma awaits elucidation. We are interested in exploring toxoplasma lanthionine synthetase C-like protein 1, which is homologous to bacterial enzymes that are responsible for prokaryotic lantibiotic synthesis, in order to investigate its ability to catalyze lanthionine formation and/or to bind TgCBS in the parasite^42^.

Even though there are several aspects regarding TgCBS and, more in general, on cysteine biosynthesis in *T. gondii * that remain unclear and deserve future investigation, this study establishes the presence of an intact functional reverse transsulfuration pathway in the parasite and demonstrates the crucial role of TgCBS in biogenesis of H_2_S. Unfortunately, there is a substantial lack of understanding about the significance of H_2_S in physiology and pathogenesis of infectious agents like *T. gondii*, even if a biological importance has been ascribed to the production of H_2_S and cysteine in almost all organisms. Interestingly, a gene encoding a putative cysteine synthase (CS) is present in the genome of *T. gondii*, therefore the parasite may be reasonably expected to possess two independent pathways for cysteine production, i.e., the reverse transsulfuration and sulphur assimilation routes. The requirement of multiple cysteine-processing alternatives in *T. gondii* may ensure high reducing power for reactive oxygen species detoxification in the host. Indeed, cysteine contributes to maintenance of cellular redox equilibrium, being the immediate precursor of glutathione, H_2_S and taurine, all of which are critical antioxidants. It is well established that the antioxidant defense system plays a key role in the host-parasite interaction for intracellular parasites, promoting their protection against the host-derived oxidative stress microenvironment. Recent studies have suggested that bacterial-derived H_2_S represents a defense system against oxidative stress and antibiotics^43^. The H_2_S signaling presumably plays a crucial role by interfering with the redox-based events even in virus-infected host cells^44^.

In the future, it will be crucial to understand whether the expression of CBS, CGL, and the putative CS might be developmentally regulated in *T. gondii*. Along with this, the generation of knockout mutants for these key genes should help to answer many open questions.

Clearly, the effects of the parasite on the redox physiology of the host and how these events could be exploited for therapeutic purposes is a challenging area of research. Undoubtedly, apicomplexa are highly vulnerable to oxidative stress, thus selective interference with the redox homeostasis of these parasites represents a promising approach for drug target.

## Materials and methods

### Chemicals

All chemicals were purchased from Sigma, unless otherwise stated.

### Production of recombinant TgCBS

Synthetic gene (Invitrogen Corporation) corresponding to the complete cDNA of TgCBS (TGME49_259180) with a tag of six His at the N-terminal was cloned into pET21a expression vector using NdeI and BamHI as restriction sites. The plasmid was used to transform *E. coli* Rosetta (DE3) expression host cells (Novagen). Cell were grown to exponential phase in LB broth at 37 °C, induced with 0.5 mM isopropyl-β-d-thiogalactopyranoside and grown for 15 h at 24 °C. Cells were pelleted by centrifugation (5,000*g* for 15 min at 4 °C) and resuspended in 20 mM sodium phosphate pH 8.5, 150 mM NaCl, 0.1 mM DTT buffer in the presence of protease inhibitor EDTA free. Cells were then disrupted by sonication on ice and centrifugated (30,000*g* for 30 min at 4 °C). The filtered supernatant was directly applied to Ni-sepharose column (GE-Healthcare) equilibrated with 20 mM sodium phosphate at pH 8.5, 150 mM NaCl, 0.1 mM DTT, and 10 mM imidazole, and eluted with the same buffer containing 500 mM imidazole. Purified TgCBS was washed in 20 mM sodium phosphate, 150 mM NaCl, 0.1 mM DTT buffer, pH 8.5, using Vivaspin concentrators (Sartorius) to remove imidazole. The homogeneity and purity of the enzyme was verified by SDS-PAGE to be > 95%. Protein was aliquoted and stored at − 80 °C until use. The yield was ~ 50 mg of protein/liter of cell culture. The cofactor (PLP) content was measured by treating TgCBS with 0.1 M NaOH and monitoring absorbance at 388 nm (ɛ_388_ = 6,600 M^−1^ cm^−1^) ^45,46^.

### Preparation of  apo-protein and determination of equilibrium dissociation constant for PLP

PLP was removed from TgCBS as a phenylhydrazone as described^31^. The obtained apo-protein showed no visible absorbance between 320–500 nm and full recovery of activity after addition of exogenous PLP.

The dissociation constant for PLP (*K*_d_) was obtained by monitoring the change of intrinsic fluorescence (excitation was set at 295 nm) of the apo-protein (1 µM) at different concentrations of PLP (0.01–5 µM) in 20 mM sodium phosphate pH 8.5, at 25 °C.

### Steady-state enzyme assays

#### CBS coupled-coupled assay

A continuous, spectrophotometric, coupled-coupled enzyme assay was used to monitor canonical TgCBS β-replacement activities. Cystathionine β-lyase and lactate dehydrogenase (CBL/LDH) were employed as auxiliary enzymes using a protocol previously described^33^ with the following modifications. The coupling enzymes were commercial LDH from rabbit muscle and recombinant CBL from *C. diphtheriae,* which is routinely used in our laboratory^31^. Reactions were performed at 37 °C in a volume of 0.2 mL in assay buffer (50 mM MOPS, 50 mM bicine, 50 mM proline (MBP) pH 9 and 20 μM PLP) in the presence of 0.2 mM NADH, 2 μM LDH, 1.5 μM CBL, 0.1–10 mM l-Ser (or 1–100 mM l-OAS) and 0.1–10 mM l-Hcys. The reactions were initiated by the addition of 0.2–2 μM TgCBS, and the oxidation of NADH to NAD^+^ was followed at 340 nm (ε_340_ = 6,200 M^−1^ cm^−1^). For each sample a background rate for all components was recorded before adding TgCBS. The reactions were performed also in the presence of SAM at different concentrations (0–0.5 mM).

For gradual thermal denaturation, TgCBS was incubated at temperatures between 30 and 70 °C for 10 min, cooled on ice for 5 min, and then residual enzymatic activity toward l-Ser was assayed in buffer containing 0.2 mM NADH, 2 μM LDH, 1.5 μM CBL, 0.8 mM l-Hcys, and 10 mM l-Ser.

#### H_2_S production assay

The production of H_2_S was determined following the formation of lead sulfide at λ = 390 nm (ε_390_ = 5,500 M^−1^ cm^−1^) upon the reaction of H_2_S with lead acetate^7^. Enzyme activity was measured in a volume of 0.4 mL at 37 °C. Reaction mixture contained 50 mM Hepes pH 7.4, 20 μM PLP, 0.4 mM lead (II) acetate, and different concentrations of l-Cys and l-Hcys (L-Hcys was not included in the l-Cys β-elimination/β-replacement reaction). The reaction was initiated by addition of 0.5–1 μM TgCBS enzyme.

The H_2_S-forming TgCBS condensation of l-Cys + l-Hcys was also examined in the presence of l-Ser as competing substrate. The substrate competition assay was performed using increasing concentration of l-Ser (0–100 mM) and fixed concentrations of l-Cys (20 mM) and l-Hcys (0.8 mM). The production of H_2_S was determined by the above described lead acetate method.

#### Data fitting and statistical analysis

Data fitting was carried out with OriginPro8 (OriginLab) software. The kinetic experiments were performed at least in triplicate using independently purified protein batches, and reported values represent means ± standard error of the mean (s.e.m.).

Kinetic parameters for TgCBS-catalyzed two-substrate reactions via ping-pong mechanism (condensation of l-Ser and l-Hcys, condensation of l-OAS and l-Hcys, and condensation of l-Cys and l-Hcys, see reactions 1, 2, 5, respectively, in Fig. 1a) were calculated from the fit of the data to the following equation:

1 $$\frac{v}{E}=\frac{{k}_{cat}\times \left[\mathrm{SA}\right]\times \left[\mathrm{SB}\right]}{{\mathrm{K}}_{\mathrm{m}}^{\mathrm{SB}}\times \left[\mathrm{SA}\right]+ {\mathrm{K}}_{\mathrm{m}}^{\mathrm{SA}}\times \left[\mathrm{SB}\right]\times \left(1+ \frac{\left[\mathrm{SB}\right]}{{\mathrm{K}}_{\mathrm{i}}^{\mathrm{SB}}}\right)+\left[\mathrm{SA}\right]\times \left[\mathrm{SB}\right]}$$

in which v is the initial velocity, E is the concentration of the enzyme, SA is the concentration of the first substrate, SB the concentration of the second substrate, *k*_cat_ and K_m_ are the catalytic and the Michaelis-Menten constants, respectively.

In canonical condensation of l-Ser (or l-OAS) and l-Hcys and the H_2_S-generating reaction between l-Cys and l-Hcys, substrate inhibition at high concentration of l-Hcys was observed. Thus, the equation included a K_i_^SB^ value, which represents the inhibition constant for substrate inhibition by l-Hcys^4,33^.

Data for H_2_S production from l-Cys by CBS was fitted following the kinetic models described in Ref.^8^.

Briefly, H_2_S production from l-Cys is the sum of two possible reactions, the β-elimination of l-Cys to generate l-Ser or the condensation of two molecules of l-Cys to generate lanthionine (reaction 2 and 3 in Fig. 1a). Data for H_2_S production from l-Cys (lead acetate assay) was fitted using Eq. (2) where v_L-ser_ and v_Lanth_ are defined by Eqs. (3) and (4), respectively^8^

2 $${v}_{H2S}={v}_{L\mbox{-}Ser}+ {v}_{Lanth},$$

3 $${v}_{L\mbox{-}Ser}=\frac{{v}_{max1 }[L\mbox{-}Cys]}{{K}_{m1(L\mbox{-}Cys)}+[L\mbox{-}Cys](1+\frac{\left[L\mbox{-}Cys\right]}{{K}_{i}})},$$

4 $${v}_{Lanth}=\frac{{v}_{max2} [L\mbox{-}Cys]{[L\mbox{-}Cys]}^{n}}{\left[L\mbox{-}Cys\right]{\left[L\mbox{-}Cys\right]}^{n}+{K}_{m1 }{[L\mbox{-}Cys]}^{n}+\left[L\mbox{-}Cys\right]{K}_{m2}^{n}},$$

where K_m1_ and V_max1_ are associated to the unimolecular reaction, K_m2_ and V_max2_ to substrate binding at the second site and the reaction velocity of the bimolecular reaction and n represents Hill coefficient.

### High-performance liquid chromatography (HPLC)

HPLC was used to identify the reaction products in the canonical and H_2_S-generating alternative reactions performed by TgCBS. HPLC analysis was performed on a Jasco LC-4000 HPLC system with a FP-4020 fluorescence detector.

The production of l-Cth form the condensation of l-Ser (or l-OAS or l-Cys) and l-Hcys was tested by incubating 2 µM TgCBS with 1 mM l-Ser (or l-OAS or l-Cys) and 0.8 mM l-Hcys. The production of l-Ser and/or lanthionine was assessed using only l-Cys as substrate (1–30 mM). Each reaction mixture was incubated for 2 h at 37 °C in 50 mM MBP pH 9.0 for canonical reactions and in 50 mM Hepes pH 7.4 for H_2_S-generating reactions. The enzyme was removed by centrifugation using the Vivaspin Turbo centrifugal concentrator (10 kDa cutoff, Sartorius). Sample solutions (120 µL) were mixed with the ortho-phthaldialdehyde (OPA) derivatization reagent to a 180 μL final volume. OPA was freshly prepared as a stock solution (25 mg of OPA, 20 µL of 2-mercaptoethanol, 0.5 mL of methanol and 4.5 mL of 0.2 M K_2_CO_3_ pH 9.6). Following a 2-min reaction at 4 °C, 10 µL of the mixture was injected onto a C-18 column (Agilent Purospher RP-18, 5 µm, 4 × 250 mm) and the derivatized products were eluted at a flow rate of 1 mL/min at 37 °C using a gradient elution with buffers A (80% 0.1 M sodium acetate pH 4.75 and 20% methanol) and B (20% 0.1 M sodium acetate pH 4.75 and 80% methanol) as described previously^7^. The fluorescence detector was set at 340 nm and 450 nm for excitation and emission wavelengths, respectively.

### Spectroscopic measurements

Absorption spectra were collected on a Jasco V-560 UV-visible spectrophotometer in 20 mM sodium phosphate pH 8.5.

CD spectra were recorded on CD spectropolarimeter (Jasco J-1500), equipped with a Peltier-type temperature controller, as previously described^47–49^. Briefly, near UV-visible (250–600 nm) spectra of 1 mg/mL TgCBS were collected in 1-cm path length quartz cuvette at a scan speed of 50 nm/min in 20 mM sodium phosphate pH 8.5 at 25 °C. A minimum of three accumulations were made for each scan, averaged and corrected for the blank solution of corresponding buffer.

Thermal denaturation profiles were collected by measuring ellipticity signal at 222 nm in a temperature range between 20 and 90 °C (scan rate 90 °C/h). Protein concentrations was 0.2 mg/mL and measurements were performed using quartz cuvettes with a path length of 0.1 cm.

Fluorescence emission spectra were recorded on a Jasco FP-8200 spectrofluorometer upon Trp excitation at 295 nm. Protein concentration varied from 1 to 5 μM in 20 mM sodium phosphate pH 8.5. Blank spectra were subtracted from sample spectra.

### Oligomeric state determination

The oligomeric state of TgCBS was investigated via gel filtration on a GE Healthcare Superdex 200 10/30 GL column in 20 mM sodium phosphate buffer pH 8.5, 150 mM NaCl and 0.1 mM DTT. High molecular weight gel filtration calibration kit (GE Healthcare) was used to construct a calibration curve, following protocols in Refs.^50,51^.

### Protein domain distribution and similarities

The protein domain distribution of CBSs derives from the analysis of the corresponding amino acid sequences with SMART^52^, the annotation in Uniprot^53^ and is guided by the crystal structures of HsCBS^1, 2^, DmCBS^5^, ScCBS^26^, and AmCBS^3^.

## Supplementary information

Supplementary Information.
