# Supplementary material for: Cystathionine β-synthase is involved in cysteine biosynthesis and H2S generation in Toxoplasma gondii
Source: Sci Rep. 2020 Sep 4;10:14657. doi: 10.1038/s41598-020-71469-x (PMC7474069; doi:10.1038/s41598-020-71469-x)
Supplement: Supplementary file 1 — Supplementary Information. [file 41598_2020_71469_MOESM1_ESM.pdf]

SUPPLEMENTARY INFORMATION

Cystathionine β-synthase is involved in cysteine biosynthesis and H_2S generation in *Toxoplasma gondii*

Carolina Conter[1†], Silvia Fruncillo[1a†], Carmen Fernández-Rodríguez[2], Luis Alfonso Martinez-Cruz[2], Paola Dominici[1] and Alessandra Astegno[1]*

[1]Department of Biotechnology, University of Verona, Strada Le Grazie 15, 37134 Verona, Italy.

[2]Center for Cooperative Research in Biosciences (CIC bioGUNE), Basque Research and Technology Alliance (BRTA), Bizkaia Technology Park, Building 801A, 48160 Derio, Spain

[a] Present address: Manchester Institute of Biotechnology, University of Manchester, 131 Princess Street, Manchester M1 7DN, United Kingdom.

[†]These authors contributed equally to the work.

*Corresponding author: Alessandra Astegno, e-mail: alessandra.astegno@univr.it

Table S1. Steady-state kinetic parameters of TgCBS for canonical reactions in the presence of SAM[a].

	**- SAM**	**+ SAM 0.5 mM**
**L-Ser + L-Hcys→L-Cth + H_2O**		
k_{cat} (s^{-1})	6.3 ± 0.4	5.9 ± 0.2
K_m $^{L\text{-}Ser}$ (mM)	0.42 ± 0.04	0.47 ± 0.03
K_m $^{L\text{-}Hcys}$(mM)	0.23 ± 0.03	0.28 ± 0.04
K_i $^{L\text{-}Hcys}$ (mM)	1.0 ± 0.1	1.1 ± 0.2
**L-OAS + L-Hcys→L-Cth + H_2O**		
k_{cat} (s^{-1})	5.5 ± 0.1	5.7 ± 0.3
K_m $^{L\text{-}OAS}$ (mM)	1.3 ± 0.2	1.6 ± 0.2
K_m $^{L\text{-}Hcys}$ (mM)	0.20 ± 0.05	0.24 ± 0.04
K_i $^{L\text{-}Hcys}$ (mM)	1.4 ± 0.2	1.7 ± 0.3

[a] Reactions were carried out in MBP buffer pH 9 containing 0.2 mM NADH, 2 μM LDH, 1.5 μM CBL, and 0.1-10 mM L-Ser (or 1-100 mM L-OAS), 0.1-10 mM L-Hcys and 0.2-2 μM TgCBS at 37°C. Data were fit to Eq. (1). Data are mean ± s.e.m.

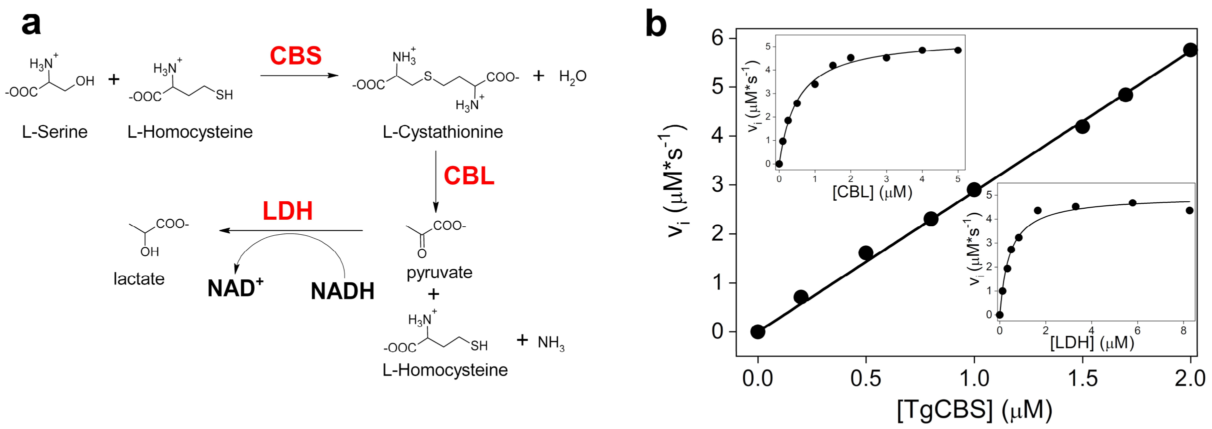

Figure S1. Coupled-coupled CBS assay. (a) Scheme of CBL/LDH coupled-coupled assay for CBS. CBS catalyzes condensation of L-Ser with L-Hcys to form L-Cth which is then converted to pyruvate, L-Hcys, and ammonia by CBL. Next, the conversion of pyruvate to lactate by LDH is accompanied by NADH oxidation, which is followed spectrophotometrically at 340 nm. **(b)** Dependence of the rate of NADH oxidation in the CBL/LDH assay as a function of TgCBS, CBL, and LDH concentration. Reactions were carried out in MBP pH 9 containing 0.2 mM NADH, 20 μM PLP, 1 mM L-Ser, 0.8 mM L-Hcys, and: *main panel*, 0.2-2 μM TgCBS, 1.5 μM CBL and 2 μM LDH; *upper inset*, 1.5 μM TgCBS, 0.1-5 μM CBL and 2 μM LDH; *lower inset*, 1.5 μM TgCBS, 1.5 μM CBL and 0.1-8.2 μM LDH.

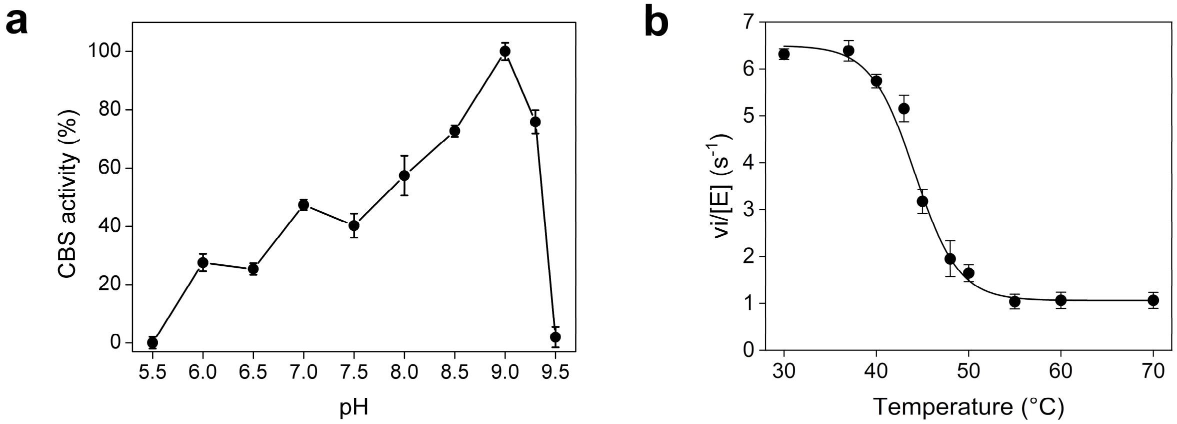

Figure S2. Effect of pH and temperature on CBS canonical activity of TgCBS. (a) pH-dependent activity profile for TgCBS performed at constant substrate concentrations (10 mM L-Ser and 0.8 mM L-Hcys) in the pH range of 5.5-9.5 at 37 °C. **(b)** Effect of thermal pre-treatment of TgCBS (10 min at temperatures between 30°C and 70°C) on its steady-state initial velocity at saturating concentration of L-Ser and L-Hcys in the canonical reaction. Each data set is relative to four replicas and error bars represent s.e.m.
